# COVID-19 Positive Cohort Undergoing Cardiac Surgery: A Possible Un(3H)oly Trinity of Hypoxia-Hemolysis-Hyperinflammation

**DOI:** 10.21470/1678-9741-2021-0077

**Published:** 2022

**Authors:** Rohan Magoon, Ramesh Kashav, Jes Jose, Ashish Walian, Souvik Dey

**Affiliations:** 1 Department of Cardiac Anaesthesia, Atal Bihari Vajpayee Institute of Medical Sciences (ABVIMS) and Dr. Ram Manohar Lohia Hospital, Baba Kharak Singh Marg, New Delhi, India.; 2 Department of Anaesthesia, Atal Bihari Vajpayee Institute of Medical Sciences (ABVIMS) and Dr. Ram Manohar Lohia Hospital, Baba Kharak Singh Marg, New Delhi, India.

**Keywords:** Covid-19, SARS-CoV-2, Cardiopulmonary Bypass, Hemolysis, Comprehension, Hypoxia

## Abstract

While the fraternity continues to ponder on the mechanisms by which coronavirus disease (COVID-19) positivity affects the outcome of cardiac surgical subset, we put forth a 3H (Hypoxia-Hemolysis-Hyperinflammation) trilogy aimed at elucidating the liaison between cardiopulmonary bypass (commonly employed for cardiac surgical conduct) and COVID-19 infection. A sound comprehension of the same can doubtlessly assist the perioperative team in staging a well-directed pathophysiology-driven management approach.

**Table:** Abbreviations, acronyms & symbols

ARDS	= Acute respiratory distress syndrome
CPB	= Cardiopulmonary bypass
COVID-19	= Coronavirus disease
DIC	= Disseminated intravascular coagulation
HIF	= Hypoxia-inducible factor
IL-6	= Interleukin-6
LDH	= Lactate dehydrogenase
PPE	= Personal protective equipment
NFκB	= Nuclear factor kappa B
SARS-CoV-2	= Severe acute respiratory syndrome coronavirus 2
TNF-α	= Tumour necrosis factor alpha

## INTRODUCTION

Amidst reports of poor perioperative outcomes in cardiac surgical patients ailing from severe acute respiratory syndrome coronavirus 2 (SARS-CoV-2) pneumonia^[1^-^3]^, we feel motivated to put forth a 3H (Hypoxia-Hemolysis-Hyperinflammation) trilogy explaining the possible perils of an interaction between cardiopulmonary bypass (CPB, commonly employed for cardiac surgical conduct) and coronavirus disease (COVID-19) infection.

While the 3Hs constitute an old challenge for perioperative cardiac practice, the pathophysiology related to COVID-19 provides new linking mechanisms to promote an enhanced crosstalk between the 3Hs. Needless to say, the aforementioned intensifies the pre-existing challenges with every likelihood of this trinity to be at the heart of the resultant organ dysfunction and subsequent morbidity and mortality in COVID-19 positive cardiac surgical cohort.

**Fig. 1:**
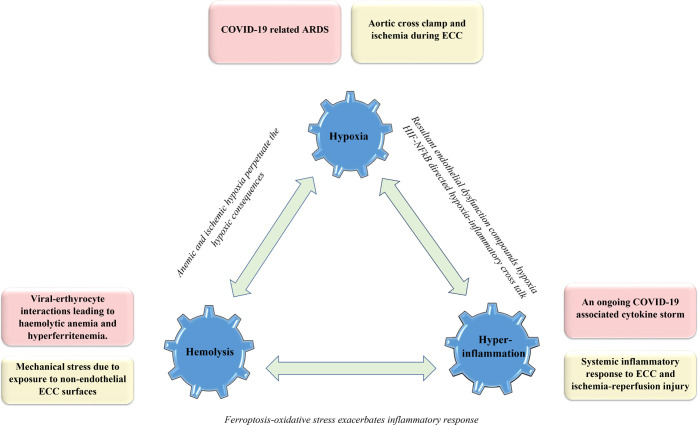
Illustration of the 3H (Hypoxia-Hemolysis-Hyperinflammation) trimodal insult owing to a double hit by COVID-19 (represented in pink) and CPB (represented in yellow). ARDS=acute respiratory distress syndrome; CPB=cardiopulmonary bypass; HIF=hypoxia-inducible factor; NFκB=nuclear factor-kappa B

Figure 1 illustrates the common links of COVID-19 and CPB3H insult alongside the role of possible interconnections in accentuating the consequences of the underlying double hit trimodal insult.

### CPB 3H COVID-19: A Double-Trouble Situation

#### I. Hypoxia at the tissue level

Given a prevailing hypoxemic milieu and an inadequate tissue oxygenation inthe setting of COVID-19-related acute respiratory distress syndrome (ARDS)^[4]^, cardiopulmonary bypass (CPB) associated microcirculatory alterations (in background of a non-pulsatile perfusion) are expected to be rather poorly tolerated at the tissue level despite an improved and controlled oxygenation on CPB^[5]^. Moreover, CPB-related hemodilution makes the matter even worse. Whileprolonged aortic cross-clamp and CPB times translate into ischemic tissue burden, the concerns about circulatory arrest (mandatory for complex open-heart surgeries) become manifold^[5^,^6]^.

#### II. Hemolysis

On one hand, mechanical shear stress and exposure to non-endothelial CPB surfaces predispose to hemolysis^[7]^. On the other hand, plausible viral-erythrocytic interactions are equally conducive to an aggravated red blood corpuscular lysis^[7]^. The present understanding of these interactions reveals that ACE2, CD26 and CD147 erythrocytic receptors serve as potential attachment sites for SARS-CoV-2 coupling, promoting hemolysis in turn by allowing the virus to mount an attack on the 1-beta haemoglobin chain. At the same time, the hepcidin-mimicking attribute of SARS-CoV-2 augments the level of circulating tissue ferritin alongside serum iron deficiency and anemia^[8]^.

#### III. Hyperinflammation

Stage III or the hyperinflammatory phase of COVID-19 infection is associated with a significant immuno-inflammatory response or cytokine storm with a marked elevation of inflammatory markers such as interleukin-6 (IL-6), C-reactive protein (CRP), lactate dehydrogenase (LDH), tumour necrosis factor alpha (TNF-α), etc.^[9^,^10]^. Focusing on the conduct of CPB in COVID-19 infected patients, the inexorable systemic inflammatory response to CPB^[10^-^12]^ and the concomitant ischemia-reperfusion injury can compound the ongoing SARS-CoV-2 cytokine storm with potentially detrimental outcomes^[13^,^14]^.

### The 3H Liaison

While the anemic hypoxia owing to hemolysis understandably perpetuates the ongoing hypoxic consequences staging obvious interactions between the Hemolysis-Hypoxia facets of the 3H liaison, other intricate molecular mechanisms associate the Hypoxia-Hyperinflammation facets wherein hypoxia-inducible factor (HIF) is closely linked to the nuclear factor kappa B (NFκB) inflammatory pathway^[15]^. In turn, the inflammatory endothelial dysfunction or endothelitis, particularly in the pulmonary microvasculature, accentuates hypoxemia^[16^,^17]^. Despite a widespread endothelitis at the cornerstone of systemic thrombotic consequences, description of an overwhelming thrombosis in the lungs compared to the whole body in COVID-19 patients has paved the way for a neoteric proposition of coining this underlying phenomenon as a ‘pulmonary intravascular coagulopathy’ in contrast to the usual nomenclature of disseminated intravascular coagulation (DIC)^[18]^. In addition to the thrombotic sequelae, the inflammatory contribution to pulmonary arterial hypertension also adds to the hypoxic predilection^[19]^. With respect to the Hemolysis-Hyperinflammation crosstalk, the hyperferritenemia and the resultant ferroptosis lead to a considerable oxidative stress that can potentially exasperate the prevailing systemic inflammatory state (Figure1)^[8]^.

### General Surgical Concerns

Health care professionals currently face peculiar challenges in the operating room and intensive care unit. Establishment of a definitive airway, suctioning, noninvasive and positive pressure mask ventilation, and supraglottic airway devices are considered risk factors for high aerosol production during surgery and post-operative care^[20]^. Moreover, establishment of a surgical airway (tracheostomy, cricothyrotomy) requires additional precautions and protective measures to avoid aerosolization, such as use of personal protective equipment (PPE) kit, advancement of endotracheal tube before puncturing the cricothyroid membrane, use of a cuffed tube, holding ventilation (if possible) when the trachea is open and compulsory use of a heat and moisture exchange filter^[21^,^22]^. This is aggravated in the cardiac surgical set-up owing to a prolonged post-operative mechanical ventilation and frequent requirement of suctioning, increasing the risk of disease spread.

On the other hand, the application of positive end-expiratory pressure commonly employed in the treatment of COVID-19 patients can negatively interact in patients after heart surgery, impairing right ventricular output and accentuating left ventricular diastolic dysfunction^[23]^. Similarly, an increased systemic inflammatory response, endothelial dysfunction and coagulopathy inexorably associated with cardiac surgical patient subset become even more relevant in COVID-19 patients (pre-existing hyperinflammation and hypercoagulopathy) aggravating the risk of end-organ dysfunction such as stroke and bleeding diathesis^[24^,^25]^.

Therefore, stratifying patients according to disease acuity, time permitted for preoperative testing for COVID-19, a judicial use of personal protective equipment kits and precautions to minimizing the exposure risk to the health care system has been advised by the American Heart Association and the American College of Surgeons amidst this fearsome pandemic era^[26]^.

## CONCLUSION

In the context of the ongoing viral pandemic, it is imperative to reconsider a strengthened and well-aligned perioperative anti-inflammatory armamentarium, rheological preservation on CPB closely backed by sophisticated tissue oxygenation monitoring, improved organ perfusion and ultrafiltration strategies on CPB, and the incorporation of advancements in CPB such as biocompatible circuits with miniaturized designs, in order to mount a concerted endeavour to combat the unholy trinity of Hypoxia-Hemolysis-Hyperinflammation in our high-risk cardiac surgical patients suffering from COVID-19. The importance of the aforementioned discussion is heralded in the title of an Editorial by Seelhammer etal.^[27]^ in a leading cardiothoracic and vascular anesthesia journal where they even consider a life-saving modality of extracorporeal membrane oxygenation (ECMO) in COVID-19 as anunhappy marriage of endothelial dysfunction and hemostatic derangements.

**Table:** Authors' roles & responsibilities

RM	Substantial contributions to the conception or design of the work; or the acquisition, analysis or interpretation of data for the work; drafting the work or revising it critically for important intellectual content; final approval of the version to be published
RK	Drafting the work or revising it critically for important intellectual content; final approval of the version to be published
JJ	Substantial contributions to the conception or design of the work; or the acquisition, analysis or interpretation of data for the work; final approval of the version to be published
AW	Drafting the work or revising it critically for important intellectual content; final approval of the version to be published
SD	Substantial contributions to the conception or design of the work; or the acquisition, analysis or interpretation of data for the work; drafting the work or revising it critically for important intellectual content; final approval of the version to be published

## References

[1] Katsiampoura A, Perozo C, Varkaris A, Vellayappan S, Tam MZ, Vellayappan U (2020). Covid-19 positivity affects outcome of cardiac surgical
patients. J Card Surg.

[2] Damodaran S, Joshi SS, Kumar V S, Natarajan P, Patangi SO, Kumaran T (2021). COVID convalescence-A boon or bane in cardiac surgery?: a "second
hit" hypothesis. J Cardiothorac Vasc Anesth.

[3] Magoon R, Kaur Kohli J, Kashav R, ItiShri (2021). Postoperative inflammation to "hyper"-inflammation: cryptic
COVID-19 connections!. Paediatr Anaesth.

[4] Magoon R (2020t). COVID-19 and congenital heart disease: cardiopulmonary
interactions for the worse!. Paediatr Anaesth.

[5] Biedrzycka A, Kowalik M, Pawlaczyk R, Jagielak D, Świetlik D, Szymanowicz W (2016). Aortic cross-clamping phase of cardiopulmonary bypass is related
to decreased microvascular reactivity after short-term ischaemia of the
thenar muscle both under intravenous and volatile anaesthesia: a randomized
trial. Interact Cardiovasc Thorac Surg.

[6] Magoon R, Kaushal B, Jose J, Kashav R (2021). Predicting lactate elevation in neonatal cardiac surgery: can the
sugars be overlooked?. J Cardiothorac Vasc Anesth.

[7] Magoon R, Dey S, Walian A, Kashav R (2020). Nitric oxide: renoprotective in cardiac surgery!. Braz J Cardiovasc Surg.

[8] Cavezzi A, Troiani E, Corrao S (2020). COVID-19: hemoglobin, iron, and hypoxia beyond
inflammation. A narrative review. Clin Pract.

[9] Siddiqi HK, Mehra MR (2020). COVID-19 illness in native and immunosuppressed states: a
clinical-therapeutic staging proposal. J Heart Lung Transplant.

[10] Magoon R, Jain A (2021). Haematological inflammatory prognostication in COVID-19: points
to ponder!. Am J Emerg Med.

[11] Magoon R, Loona M, Kohli JK, Kashav R (2020). Cytokine adsorption in cardiac surgery: where do we
stand?. Braz J Cardiovasc Surg.

[12] Magoon R, Makhija N, Das D (2019). Vasoplegic syndrome after cardiac surgery: better the devil you
know!. J Card Surg.

[13] Dey S, Kashav R, Kohli JK, Magoon R, Walian A, ItiShri (2021). Systemic immune-inflammation index predicts poor outcome after
elective off-pump CABG: a retrospective, single-center study. J Cardiothorac Vasc Anesth.

[14] Magoon R, Makhija N (2020). Endothelial glycocalyx and cardiac surgery: newer
insights. J Cardiothorac Vasc Anesth.

[15] Biddlestone J, Bandarra D, Rocha S (2015). The role of hypoxia in inflammatory disease
(review). Int J Mol Med.

[16] Magoon R, Kohli JK, Kashav R, ItiShri (2021). Inhaled milrinone for sick COVID-19 cohort: a pathophysiology
driven hypothesis!. Med Hypotheses.

[17] Magoon R (2021). Pulmonary vasculature in COVID-19: mechanism to
monitoring!. Korean J Anesthesiol.

[18] Belen-Apak FB, Sarıalioğlu F (2020). Pulmonary intravascular coagulation in COVID-19: possible
pathogenesis and recommendations on anticoagulant/thrombolytic
therapy. J Thromb Thrombolysis.

[19] Magoon R (2021). The pulmonary circuit dynamics in COVID-19!. J Anesth.

[20] Di Saverio S, Pata F, Khan M, Ietto G, Zani E, Carcano G (2020). Convert to open: the new paradigm for surgery during
COVID-19?. Br J Surg.

[21] Lima DS, Ribeiro MF, Vieira-Jr HM, Campos T, Saverio SD (2020). Alternatives for establishing a surgical airway during the
COVID-19 pandemic. Rev Col Bras Cir.

[22] Yánez Benítez C, Güemes A, Aranda J, Ribeiro M, Ottolino P, Di Saverio S (2020). Impact of personal protective equipment on surgical performance
during the COVID-19 pandemic. World J Surg.

[23] Magoon R (2021). Left-ventricular diastolic dysfunction in coronavirus disease:
opening Pandora's box!. Korean J Anesthesiol.

[24] Magoon R, Bansal N, Singh A, Kashav R (2021). Methylene blue: subduing the post COVID-19 blues!. Med Hypotheses.

[25] Magoon R (2021). COVID-19 related strokes: Pandora's Box may open as the p(c)lot
thickens!. Neurologia.

[26] Patel V, Jimenez E, Cornwell L, Tran T, Paniagua D, Denktas AE (2020). Cardiac surgery during the coronavirus disease 2019 pandemic:
perioperative considerations and triage recommendations. J Am Heart Assoc.

[27] Seelhammer TG, Plack D, Lal A, Nabzdyk CGS (2020). COVID-19 and ECMO: an unhappy marriage of endothelial dysfunction
and hemostatic derangements. J Cardiothorac Vasc Anesth.

